# Cancer, Health Literacy, and Happiness: Perspectives from Patients under Chemotherapy

**DOI:** 10.1155/2013/291767

**Published:** 2013-09-05

**Authors:** Sara Maria Oliveira Pinto, Sílvia Maria Alves Caldeira Berenguer, José Carlos Amado Martins

**Affiliations:** ^1^Abel Salazar Biomedical Sciences Institute, University of Porto, Rua de Jorge Viterbo Ferreira No. 228, 4050-313 Oporto, Portugal; ^2^Catholic University, Palma de Cima, 1649-023 Lisbon, Portugal; ^3^Medical-Surgical Unit, Nursing School of Coimbra, Avenida Bissaya Barreto-Apartado 7001, 3046-851 Coimbra, Portugal; ^4^Department of Human Sciences and Health, Medicine Faculty, University of Oporto, Al. Hernâni Monteiro, 4200-319 Porto, Oporto, Portugal

## Abstract

Cancer is a dreaded disease that affects all dimensions of human life. In this context, issues related to the quality of life—as happiness, perception about health status, or health literacy—are important. This study aims to analyze the following topics the perception: the Portuguese cancer patients have about their health status while undergoing chemotherapy, the satisfaction with the information relating to their health, their level of happiness, and their vision of the future. An observational, cross-sectional, and descriptive study was developed. Data were collected between May and July 2012 in the day hospital of a central hospital in northern Portugal. The sample was composed of 92 cancer patients who were asked to answer a questionnaire during chemotherapy. The results indicate that, despite this life-threatening disease, patients consider themselves fairly happy and have an optimistic view of the future. Information about their health condition and religious beliefs was important coping mechanisms to help dealing with the suffering caused by the disease. The study highlights the importance of providing care in a holistic way. Nurses must be alert and available to listen, answer questions, provide supporting structures, or refer to other professionals when needed.

## 1. Introduction 

The scientific-technological development that has occurred in the last half of the twentieth century determined important changes in the course of the disease, being the pathologies of chronic character the leading cause of death in modern societies. However, this increase in longevity brought another kind of concerns related to comorbidities and limitations that arise as a result of living with a chronic illness. In general, we live longer but this increase in life expectancy does not always correspond to better quality of life [1].

In this context, issues related to quality of life have been gaining special importance and are widely studied in various contexts, particularly in oncology. Cancer—which is one of the leading causes of death worldwide—is one of the most dreaded diseases in the present [2, 3]. The strong physical, psychological, social, emotional, and spiritual impact reminds the person of one's vulnerability and leads one to wonder about the meaning of life, and that may cause suffering [4].

Recent studies have shown that the concept of quality of life is closely associated with self-perception about wellbeing, with the satisfaction with life and happiness [5]. In this context, health professionals, particularly in the context of palliative care—whose mission is to promote the best possible quality of life for the patient and family [6]—have been encouraged to investigate and find strategies that allow the person to find moments of happiness and wellbeing, even in difficult circumstances, as is the experience of a cancer or terminal disease [7].

Happiness is an essential component of quality of life, to the point that the World Health Organization recognized it as part of the concept of health [8]. However, as documented by other authors [7], little is known about its significance for people living with advanced cancer. After a comprehensive review, we found some studies where the concept was explored in patients with cancer [7, 9], Parkinson's disease [10], and Alzheimer's disease [11]. Yet none of these was held in Portugal or in the context of palliative care, where the theme is especially important. These studies are recent and emerged in the last 10 years which suggests a growing interest in the topic in the context of health. 

Previous research suggests that happiness is a complex multidimensional concept, influenced by individual and cultural factors, with strong repercussions in all dimensions of human life [7]. In a recent study, the authors concluded that happiness is perceived by patients with advanced cancer as a fundamental precursor to quality of life, helping them to live the remaining time in the best possible way [7].

Another concept that has been widely studied has been the concept of health literacy. The health literacy is defined as a person's ability to obtain, process, and understand information on health and how this process can help one in decision-making [12, 13]. The research has suggested that health literacy helps people to make informed and aware decisions, which are extremely important factors in an increasingly complex health care system [9]. In addition to these considerations, efforts have been alerting health professionals to the fact of low literacy, particularly in cancer patients, being associated with lower levels of welfare, higher levels of anxiety and depression, erroneous conceptions about the disease, and, consequently, lower adherence to the therapeutic regimen and less satisfaction with the care provided [12, 14–16].

The information given to the patient as well as the decisions to be taken, particularly in contexts of severe disease, is difficult and with repercussions, not only for professionals but also for the patient and family. In this context, nurses assume a privileged position, because the proximity to the patient allows them to be more attentive to their needs and problems [17].

Assuming that happiness and health literacy are important to patients with advanced cancer, we conducted the present study, which aims at analyzing (a) the perception Portuguese cancer patients undergoing chemotherapy have about their health status, (b) the satisfaction they have with the information relating to their health status, (c) their level of happiness, and (d) how these patients perceive their own future.

## 2. Methods

### 2.1. Research Design, Sample, and Setting

An observational, cross-sectional, exploratory, and descriptive study was developed, aiming at understanding how information about the health status and their belief systems influence the vision of the future and happiness of people undergoing chemotherapy.

Portuguese cancer patients undergoing chemotherapy were the chosen population for this study. Due to the impossibility of accessing all these patients, it was decided to restrict the study to patients who undergone chemotherapy in the day hospital (ambulatory care) of a central hospital in northern Portugal. The reason of this choice is due to the fact that this is a large department, with patients from different age groups and also with different malignancies, facts that we considered important in order to obtain a representative sample.

The following inclusion criteria were defined: (a) 18-year olds, (b) Portuguese nationality, (c) malignant disease diagnosed, and (d) undergoing chemotherapy. For ethical and legal reasons, all patients under 18 and with cognitive limitations, as well as those undergoing chemotherapy for the first time were excluded from the study.

Data were collected between May 16, 2012, and July 16, 2012, according to instructions and availability of the service where the study was conducted. In order to assure the respect for ethical principles, particularly the nonmaleficence, and so as not to interfere with the care and service dynamics, data were collected during the treatments, which generally lasted several hours. In this period all patients undergoing chemotherapy were approached (regardless of the purpose of this be curative or palliative), up to the limit of the sample under study, and according to the inclusion and exclusion criteria defined. Only 10 out of the 102 patients approached refused to participate in the study. The sample was nonprobabilistic, consecutive, and consisted of 92 cancer patients. 

### 2.2. Instruments

The data collection instrument was built by a self-completion questionnaire, consisting of two distinct parts. 

In the first part, information to sociodemographic, clinical, and faith was requested. From the data collected here, we analyzed the following variables: gender, age, marital status, education, household composition, diagnosis, time on chemotherapy, purpose of chemotherapy, religion, religious practice (practicing or not practicing), and opinion about God.

In the second part of the instrument, which resulted from an adaptation of the *Permanent Survey on Social Attitudes of the Portuguese* [15], five closed-answer questions (Likert scale) related to the perception of health status, satisfaction with the information provided on the state of health, vision of the future, fear of the future, and the level of happiness (dependent and ordinal variables) were placed. The final instrument was reviewed by an expert panel constituted by the authors and three nurses (an expert in the validation of scales, a spirituality researcher and a palliative care expert). Subsequently, we performed a pre-test with 10 cancer patients receiving chemotherapy. This pre-test concluded that the instrument was simple, clear and objective, which is why it was not necessary to make changes to it.

### 2.3. Ethical Issues and Data Collection

The research project was previously approved by the ethics committee and the administration council of that institution. All participants gave their freely informed and written participation in the study. The data were analyzed jointly, ensuring anonymity and confidentiality of responses.

The information was analyzed using the Statistical Package for the Social Sciences program, version 18 for Windows, and descriptive statistics were used.

## 3. Findings

### 3.1. Sociodemographic, Clinical, and Religious Characteristics

The participants in the study (*n* = 92) were approximately 54 years old (mean = 54.17 years). The youngest patient was 18 and the oldest 79 years. In the total of respondents, 51.10% were male and 48.90% female. Most of them lived only with spouse (46.70%) and 38.00% with the spouse and children. Despite being with a malignant disease in an advanced stage, 2 patients (2.20%) reported living alone.

Regarding the qualifications, we found that the majority of subjects had only 4 years of education (47.90%), which corresponds to the elementary education in Portugal.

From a clinical standpoint, it was found that most respondents had bowel cancer (25.29%) and breast cancer (20.70%). The average time in chemotherapy was 11.80 months. Of the total respondents, 54.30% were in chemotherapy with curative intent and 45.70% in chemotherapy with palliative goals. The majority of individuals in palliative chemotherapy were males (29.30%) and had bowel cancer (10.90%). Patients in curative chemotherapy were essentially females (32.60%) and had breast cancer (17.40%).

In the item about religious beliefs, patients were questioned about their religion, religious practice, and beliefs about God. Six patients did not respond to the questions “religion” and “religious practice.” The majority of claims to be Catholic (93.00%) and it was found that there is no great disparity between those who admit having a regular religious practice (51.10%) compared to those who do not have it (48.80%). All respondents answered the question about God's opinion. The vast majority has no doubts about God's existence (60.90%). However, those who have a regular religious practice are those with more certainty (40.50%). Only one patient reported not believing in God.

### 3.2. Patient Perspectives on Perception about the State of Health, Information, Future Vision, and Happiness

When asked how they regarded their health status at that time, most patients (51.10%) rated it as “neither good nor bad.” Despite this neutral position, 30.40% considered that their health was “good,” being relatively low the percentage of patients who consider their health as “bad” or “very bad” (13.30%) (Table 1).

With regard to how they saw their future, few have considered it “very good” (7.60%), slightly higher than that found for the group of patients who considered their health status as “very good” (4.30%).

In general, despite the difficulties imposed by the disease, the vision of the future is always more optimistic than the perception of the current state of health: 35.90% perceives the future as “good” and although 3.30% consider their health very bad, only 2.20% believe that the future will be—itself—very bad. However, the great majority is afraid of the future (63.00%) and 18.50% reported being very frightened.

With regard to the degree of satisfaction with information on the health status—and despite not having analyzed the information they had—it was found that the great majority of the patients felt very satisfied (44.60%) or satisfied (29.30%) with the information about their health status.

In the last issue, participants were encouraged to think about all aspects of their life and assess their current state of happiness. In spite of the majority considering to be “fairly happy” (57.60%), it is important to note that

- there is a significant group of patients who considered themselves to be “very happy” (23.90%);
- very few considered themselves unhappy (2.20%);
- among those “fairly happy” considered, 64.30% were in palliative chemotherapy;
- 16.70% of the patients in palliative chemotherapy considered themselves as very happy people.

Analyzing these items, the variables were crossed (Table 2).

It was found that there is a large percentage who considers their health as “good” or “very good” (35.20%), which is quite interesting considering they are individuals with a very serious disease. However, it is important to note that those who perceive their health status as “bad” or “very bad” are mostly in palliative chemotherapy (14.60%).

With regard to the vision of the future and happiness, it was found that satisfaction with information about the health status exerts an important influence. 

In general, patients who considered themselves well informed about their health status reported having no fear of the future (24.20%), and, despite living with a serious life-threatening cancer disease, they saw it as “good” (22.00%). On the other hand, when we perform the summation of the responses of those who consider themselves less informed about their health status, we found that 17.60% are afraid of the future.

Still regarding the variable “information,” it was found that those who consider themselves more informed about their health status, are also considered happier. In fact, 46.70% feel satisfied or very satisfied with the information relating to their health, considering themselves fairly happy or very happy. Those more informed about their health status, are also those who fear the future less (24.20%).

Finally, when the variables of the future vision and beliefs about God were crossed (Table 3), it was found that those who believe in God and have no doubts about His existence see the future as “neither good nor bad” (25.30%) or “good” (25.30%). Similarly, these are also the individuals who mostly refer not being afraid of the future (23.10%) or only slightly (19.80%).

## 4. Discussion

The results show us the perspective of a group of patients with advanced cancer in chemotherapy.

Previous studies have shown that cancer is one of the most feared diseases by modern societies, with strong impact on the quality of life of the human person, particularly during chemotherapy treatment [18].

Other studies have been documented that quality of life is closely linked to the perception that the person has in relation to one's wellbeing, life satisfaction, and happiness [7].

The study confirms this vision but it also adds new data, suggesting that religious beliefs and health literacy may have an important contribution in promoting wellbeing and patient's quality of life.

Although all participants have an advanced cancer, some of them in palliative chemotherapy, the vast majority has an optimistic view of the future and considers themselves as happy persons. The majority reported being afraid of the future but, despite this, their vision of the future has always been more positive than their perception about health status.

This optimistic vision is associated with hope, a concept closely associated with wellbeing and happiness and essential in the care of people with cancer. Previous studies have shown that the need to maintain a positive thinking is crucial because it helps the person to deal with the suffering caused by the disease, to restore the meaning of life and find purpose to continue [19–21].

It was also found that religious beliefs can have a significant impact on how a person views the future. Although the majority of respondents are Catholic, as fits the profile of Portuguese citizens, beliefs about God have varied. The study revealed that those who believed in a higher entity and not had any doubts about his existence had less fear of the future. These data confirm those obtained by other authors and show that religious and spiritual beliefs are closely associated with the concept of quality of life, helping the person to maintain hope, and dealing with the uncertainty of the future in a more effective way [20–22].

In addition to this positive view tendency, most respondents considered themselves happy or very happy, noting that a significant group of these patients was in palliative chemotherapy. This seems to us to be an important indicator of the quality of life of this group of patients and therefore alerts us to the importance of providing care in a holistic perspective, in order to provide patients with moments of wellbeing and comfort even at the time of disease.

Happiness is a complex and multidimensional experience and, in this sense, health professionals should strive to meet the needs of the human person in all dimensions of life: physical, psychological, social, emotional, and spiritual [7].

In addition to these data, this study has also shown that the information that the patient has about their health status and the way how one processes and perceives that information may have an important influence on how the future is looked and consequently on their happiness. We found that patients more satisfied with the information regarding their health status had less fear of the future, regard it as better, and consider themselves happier. These data are corroborated by a study carried out in 2003 with cancer patients, where the author concluded that patients who knew the effects of treatment had a more positive perception of their health status and higher levels of hope [23]. In the present study, we did not assess which information was given to participants, but we found that the information given was enough to answer their questions and doubts, helping them to live that period of their lives in the best possible way. In this context, we can say that more important than “what information to give the patient” is “how” and “when” to give this information [24]. The low health literacy—which includes not only the information given, as well as how the person understood and perceived this information—is associated with poorer health outcomes, including lower adherence to the therapeutic regimen, misconceptions about the disease, and higher levels of depression and anxiety [12, 14–16].

The study shows, however, some limitations. The sample type (not randomized) does not allow the inference of the results for the population. Moreover, the nationality of the participants in the study may have influenced the results. Generally, the Portuguese are seen as optimistic people, who tend to face the future with hope, as suggested in previous studies [20, 21, 25]. For practical reasons, the study was limited to patients who carry out chemotherapy in day hospital. Within this group, patients undergoing chemotherapy for the first time were excluded. Although ethical motivations should prevail, it is important to note that these criteria limit the results. Thus, the data refer to the sample under study and future generalizations should be made with caution. 

Despite these limitations, the present work encourages health care professionals to find ways of helping cancer patients live the period of the disease in the best possible way. The information transmitted and how the patient processes this information, along with the religious beliefs, can act as important coping mechanisms, helping the person to deal with suffering, to look to the future with hope and be happier.

## 5. Conclusions

Although all participants in the study had severe malignant disease, some of which in palliative care, it was found that—in general—they are happy people with an optimistic view of the future.

Information on health status, commonly referred to as health literacy, plays an important role in these results, showing that patients who feel well informed, fear less the future, perceive it as better and consider themselves happier.

Despite the study's limitations, particularly with regard to the type of sampling (which is not random prevented us infer the results to the population), we believe that the results are important.

The study supports scientific evidence, particularly the idea that health literacy and promotion of happiness can have a strong impact on people's quality of life with advanced chronic disease, helping them to live the moments of crisis in the best possible way.

The study has thus important implications for future research and for clinical practice, showing the importance of providing care in a holistic manner, directed to the ill person and not just to fight the disease. We live in a time characterized by technology and that mechanized vision should never be extended to the nursing care which is holistic and human in its nature. However, this study demonstrates the importance of humanizing care. Nurses must therefore be alert and seek to collect a range of information that may be important coping mechanisms such as religious beliefs, family support, and information about the health status. Another important aspect passes through the demystification of some wrong information people have about the disease and about cancer treatments, and which may affect how a person lives in the present and sees the future. Results show that information about the state of health is not only a fundamental human right, but also an important promoter of happiness, wellbeing, and therefore quality of life. 

Cancer is the most feared disease in today's society. Thus, it is absolutely essential that nurses help fight the fear associated with the disease and treatments, trying to be available to listen, answer questions, provide supporting structures, or refer to other professionals when needed. However, simple gestures such as praying with the ill person, the therapeutic touch, listening, or teaching the family can also be extremely important in helping to reduce the patient's suffering and promoting their happiness and wellbeing.

Happiness, in turn, is determined by a set of circumstances where the health literacy and religious beliefs can play a key role.

Both concepts—health literacy and happiness—are closely associated with hope. However, more studies are needed to relate the association between these concepts and analyze the indexes of happiness based on the type of information given.

## Figures and Tables

**Table 1:** Perception of health status, vision and fear of the future, information, and happiness (*n* = 91*).

Perceived health status *How do you consider your* *state of health at the moment? *			Vision of the future *How do you see your future? *			Fear of the future *I am afraid of the future. *			Information about the state of health *I feel I am well informed on about my health.*			Happiness *Please, think in all aspects of your* *current life.* *All in all you say you feel: *		
	*n*	%		*n*	%		*n*	%		*n*	%		*n*	%
Very bad	3	3.30	Very bad	2	2.20	I do not agree	33	35.90	I do not agree	3	3.30	Not happy	2	2.20
Bad	9	22.80	Bad	15	16.30	I slightly agree	28	30.40	I slightly agree	21	22.80	Few happy	15	16.30
Neither good nor bad	47	29.30	Neither good nor bad	34	37.00	I quite agree	13	14.10	I quite agree	27	29.30	Fairly happy	53	57.60
Good	28	44.60	Good	33	35.90	I totally agree	17	18.50	I totally agree	41	44.60	Very happy	22	23.90
Very good	4	4.30	Very good	7	7.60									

*1 missing.

**Table:** (a)

		Purpose of chemotherapy		Total
		Curative	Palliative	
State of health perceptions *How do you consider your state of health at the moment? *				
*Very bad *	Count % of total	2 4.00	1 2.40	3 3.30
*Bad *	Count % of total	4 8.00	5 12.20	9 9.90
*Neither good nor bad *	Count % of total	25 50.00	22 53.70	47 51.60
*Good *	Count % of total	16 32.00	12 29.30	28 30.80
*Very good *	Count % of total	3 6.00	1 2.40	4 4.40

Total	Count % of total	50 100.00	41 100.00	91* 100.00

**Table:** (b)

		Information about the state of health *I feel I am well informed on about my health. *				Total
		*I do not agree *	*I slightly agree *	*I quite agree *	*I totally agree *	
Fear of the future *I'm afraid of the future. *						
*I do not agree *	Count % of total	1 1.10	6 6.60	4 4.40	22 24.20	33 36.30
*I slightly agree *	Count % of total	0 0.00	6 6.60	14 15.40	8 8.80	28 30.80
*I quite agree *	Count % of total	1 1.10	1 1.10	6 6.60	5 5.50	13 14.30
*I totally agree *	Count % of total	1 1.10	7 7.70	3 3.30	6 6.60	17 18.70

Total	Count % of total	3 3.30	20 22.00	27 29.70	41 45.10	91* 100.00

**Table:** (c)

		Information about the state of health *I feel I am well informed on about my health. *				Total
		*I do not agree *	*I slightly agree *	*I quite agree *	*I totally agree *	
Happiness *Please, think in all aspects of your current life. All in all you say you feel: *						
*Not happy *	Count % of total	0 0.00	1 1.10	1 1.10	0 0.00	2 2.20
*Few happy *	Count % of total	1 1.10	7 7.70	4 4.40	3 3.30	15 16.30
*Fairly happy *	Count % of total	2 2.20	8 8.80	20 21.70	23 25.00	53 57.60
*Very happy *	Count % of total	0 0.00	5 5.50	2 2.20	15 16.30	22 23.90

Total	Count % of total	3 3.30	21 22.80	27 29.30	41 44.60	92 100.00

*1 missing.

**Table 3:** Vision of the future and opinion about God.

		Opinion about God**						Total
		I	II	III	IV	V	VI	
Vision of the future *How do you see your future? *								
*Very bad *	Count % of total	0 0.00	0 0.00	0 0.00	1 1.10	0 0.00	1 1.10	2 2.20
*Bad *	Count % of total	0 0.00	0 0.00	3 3.30	3 3.30	3 3.30	6 6.60	15 16.50
*Neither good nor bad *	Count % of total	0 0.00	1 1.10	3 3.30	4 4.40	3 3.30	23 25.30	34 37.40
*Good *	Count % of total	0 0.00	2 2.20	4 4.40	2 2.20	2 2.20	23 25.30	33 36.30
*Very good *	Count % of total	0 0.00	0 0.00	2 2.20	0 0.00	2 2.20	3 3.30	7 7.70

Total	Count % of total	0 0.00	3 3.30	12 13.20	10 11.00	10 11.00	56 61.50	91 100.00

**(I) I do not believe in God. (II) I do not know if God exists or believe there is a way to know this. (III) I do not believe in a personal God, but I believe in the existence of a supreme force whatsoever. (IV) There are times when I believe in God and times when I do not. (V) In spite of my doubts, I feel that I believe in God. (VI) I know that God exists and I have no doubt about that.
